# The driver landscape of sporadic chordoma

**DOI:** 10.1038/s41467-017-01026-0

**Published:** 2017-10-12

**Authors:** Patrick S. Tarpey, Sam Behjati, Matthew D. Young, Inigo Martincorena, Ludmil B. Alexandrov, Sarah J. Farndon, Charlotte Guzzo, Claire Hardy, Calli Latimer, Adam P. Butler, Jon W. Teague, Adam Shlien, P. Andrew Futreal, Sohrab Shah, Ali Bashashati, Farzad Jamshidi, Torsten O. Nielsen, David Huntsman, Daniel Baumhoer, Sebastian Brandner, Jay Wunder, Brendan Dickson, Patricia Cogswell, Josh Sommer, Joanna J. Phillips, M. Fernanda Amary, Roberto Tirabosco, Nischalan Pillay, Stephen Yip, Michael R. Stratton, Adrienne M. Flanagan, Peter J. Campbell

**Affiliations:** 1 Cancer Genome Project, Wellcome Trust Sanger Institute, Wellcome Trust Genome Campus, Hinxton, Cambridgeshire, CB10 1SA UK; 2 Department of Paediatrics, University of Cambridge, Cambridge, CB2 0QQ UK; 3 Corpus Christi College, Cambridge, CB2 1RH UK; 4 Los Alamos National Laboratory, Los Alamos, NM 87545 USA; 5 UCL Great Ormond Street Institute of Child Health, London, WC1N 1EH UK; 6 Department of Paediatric Laboratory Medicine, The Hospital for Sick Children, Toronto, ON Canada M5G 1X8; 7 Department of Genomic Medicine, MD Anderson Cancer Center, University of Texas, Houston, TX 77030 USA; 8 University of British Columbia, Vancouver, BC Canada V6T 1Z4; 9 Bone Tumour Reference Centre, Institute of Pathology, University Hospital Basel, University of Basel, 4031 Basel, Switzerland; 10 Division of Neuropathology and Department of Neurodegenerative Disease, The National Hospital for Neurology and Neurosurgery, University College Hospital NHS Foundation Trust and UCL Institute of Neurology, London, WC1N 3BG UK; 11 Department of Pathology and Laboratory Medicine, Mount Sinai Hospital, Toronto, ON Canada M5G 1X5; 12 Chordoma Foundation, PO Box 2127, Durham, NC 27702 USA; 13 Department of Neurosurgery, University of California, San Francisco, CA 94143 USA; 14 Department of Histopathology, Royal National Orthopaedic Hospital NHS Trust, Middlesex, Stanmore, HA7 4LP UK; 15 University College London Cancer Institute, London, WC1E 6BT UK; 16 Department of Haematology, University of Cambridge, Cambridge, CB2 2XY UK

## Abstract

Chordoma is a malignant, often incurable bone tumour showing notochordal differentiation. Here, we defined the somatic driver landscape of 104 cases of sporadic chordoma. We reveal somatic duplications of the notochordal transcription factor brachyury (*T*) in up to 27% of cases. These variants recapitulate the rearrangement architecture of the pathogenic germline duplications of *T* that underlie familial chordoma. In addition, we find potentially clinically actionable PI3K signalling mutations in 16% of cases. Intriguingly, one of the most frequently altered genes, mutated exclusively by inactivating mutation, was *LYST* (10%), which may represent a novel cancer gene in chordoma.

## Abstract (web summary)

Chordoma is a rare often incurable malignant bone tumour. Here, the authors investigate driver mutations of sporadic chordoma in 104 cases, revealing duplications in notochordal transcription factor brachyury (*T*), PI3K signalling mutations, and mutations in LYST, a potential novel cancer gene in chordoma.

## Introduction

Chordoma is a rare, aggressive bone cancer showing notochordal differentiation that mainly affects adults and occasionally children, with a marked predilection for the axial skeleton^1^. The principal treatment of chordoma is radical surgical resection, augmented by adjuvant radiotherapy^2^. Chordoma does not respond to cytotoxic chemotherapy^3^. Most patients cannot be cured^3^. They often suffer from the debilitating consequences of tumour progression and its surgical treatment before dying from their disease^3^.

The genetic basis of sporadic chordoma has been investigated using copy-number arrays and targeted sequencing limited to candidate genes^4–6^. Collectively, these studies have identified recurrent loss of *CDKN2A* as a key driver in chordoma development. In addition, occasional alterations of PI3K signalling genes have been reported and evaluated in expression studies and in vitro^7–9^.

Rare familial cases of chordoma are attributable to focal germline tandem duplication of the *T* gene, which encodes the transcription factor of notochordal development, brachyury^10^. Brachyury expression is the diagnostic hallmark of chordoma^1^ and has been proposed to prevent the notochord from progressing into senescence^11^. Utilising array CGH, quantitative PCR and FISH, somatic amplification of *T* in sporadic chordoma has been demonstrated, predominantly in the context of whole-chromosome gains, rather than focal amplification^11^. However, other studies have not identified recurrent somatic copy-number gains of *T* in chordoma^4^. Furthermore, in sporadic tumours certain germline SNPs of *T* have been associated with chordoma^7, 12, 13^.

Here, we present DNA sequences of 104 cases of sporadic chordoma, divided into a discovery (*n* = 37) and a validation cohort (*n* = 67). The discovery cohort, comprising paired tumour and normal-tissue DNA, was subjected to whole-genome sequencing (*n* = 11) or whole-exome (*n* = 26) sequencing. The validation cohort, 15 paired and 52 unpaired cases, was studied by targeted sequencing of 360 cancer genes (Supplementary Data 1). Transcriptome sequencing was performed in nine of the whole-genome cases (Supplementary Data 2). Copy-number profiles were generated from SNP6 arrays or directly from sequencing reads. Somatic mutations were identified and annotated using the analysis pipeline of the Cancer Genome Project^14^. In addition, we also re-analysed whole-genome sequences of four chordoma cell lines (UM-Chor1, U-CH2, JHC7 and Mug-Chor1) that are publicly available (www.chordomafoundation.org).

## Results

### Overview of somatic changes in chordoma

**Fig. 1:**
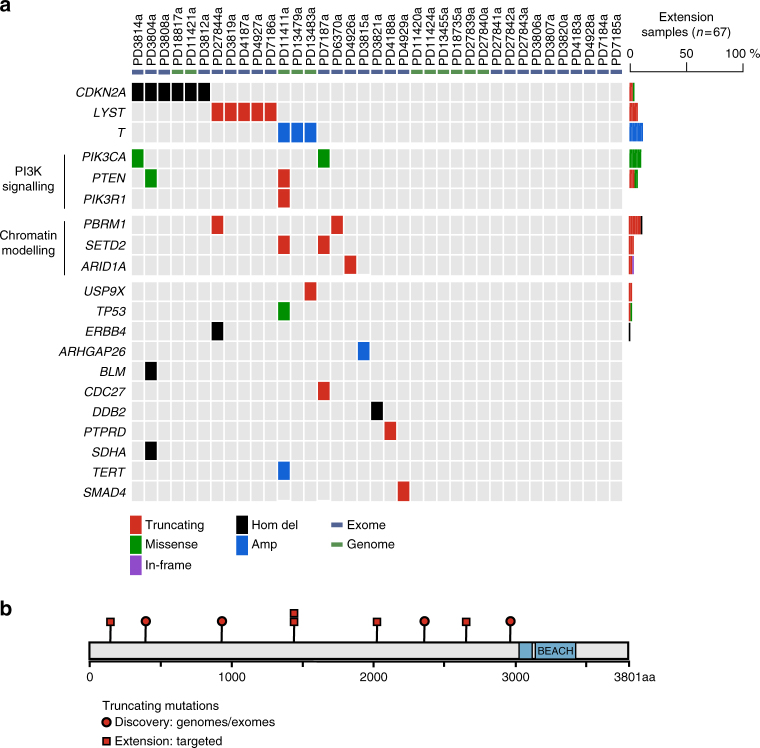
Driver landscape of 104 chordomas. **a** For the discovery cohort, in every tumour (column), driver events are indicated by gene (row) and type of mutation (colour coded; refer to legend). For extension cases, the overall prevalence of driver events in each gene is indicated (colour coded by mutation class). **b** The LYST protein is depicted, indicating the position of truncating mutations in the discovery (red circles) and extension (red squares) samples. Functional domains are indicated in blue

The overall somatic mutation burden of chordomas was modest. In the discovery cohort of genomes and exomes (*n* = 37), we identified 5 to 76 coding substitutions (median 21) and 0 to 47 indels (median 4) per case (Supplementary Data 2 and 3). The predominant substitution signature, as defined by the trinucleotide context of 27,612 substitutions found in 11 chordoma genomes, reflected two mutational processes. These are universally present across cancer types and show age-related accumulation^15^ (Supplementary Data 2 and 4). Occasional rearrangements and copy-number changes, including chromothripsis, were observed (Supplementary Data 5). No recurrent gene fusions were found in whole-genome or transcriptome sequences. Manual curation of driver variants defined the landscape of cancer genes operative in chordoma (Fig. 1a) and chordoma cell lines (Supplementary Table 1).

### Somatic duplications of *T*

**Fig. 2:**
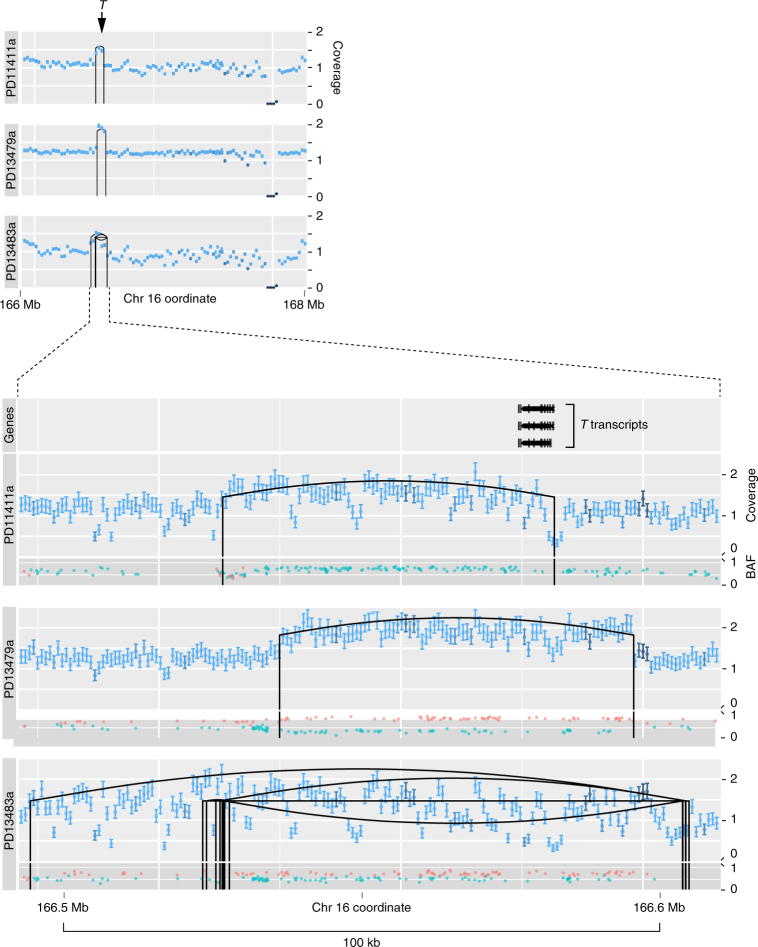
*T* duplications in 11 chordoma genomes. Coverage tracks: for each tumour, the coverage of the *T* locus is shown over a 2 Mb window (upper panel) and a 100 kb window (lower panel). *X* axis: genomic position. *Y* axis: coverage (unit: number of 5′ ends of fragments in a bin/(length of bin × reads for sample) × 3 × 10^9^). The error bars show the 95% confidence interval for the true mean coverage in each coverage bin. The colour coding of the error bars expresses the average mapping score in each bin (light = high; dark = low mapping score). Rearrangements are displayed as vertical black lines at the breakpoints connected by an upward arc for tandem duplications, a downward arc for deletions, and a straight line for inversions. BAF (B-allele frequency) track: the BAF tracks show the allele frequencies of heterozygous SNPs. Allele frequencies were plotted in a colour corresponding to their inferred parental chromosome, as determined by comparison to imputed haplotype blocks provided by the 1000 genomes project. *X* axis: genomic position. *Y* axis: B-allele frequency

In three of the eleven tumour genomes (27%), we found focal, somatically acquired duplication of the *T* gene, 70 to 136 kb in size, akin to those observed in familial cases (Fig. 2). In each case, the rearrangement mechanism underlying the gain of *T* was defined and resolved at base-pair resolution (Supplementary Table 2). In two cases (PD1141a and PD13479a), a simple tandem duplication covering at least one full length *T* transcript was evident. In the third case (PD13483a), a complex pattern of rearrangement was present resulting in the intact duplication of *T*. The remarkable focality and low-amplitude signal of the observed *T* duplications rendered them undetectable by copy-number arrays (SNP6). Consequently, we searched for the presence of additional *T* duplications in an extension cohort by designing a targeted sequencing assay, which included the entire footprint of *T* and flanking regions. Although this assay is less sensitive than whole-genome sequencing, we found additional cases with gains of *T* in the extension cohort (8/67, 12%; Supplementary Fig. 1). When confined to the most informative samples with a high density of heterozygous SNPs, *T* gains were seen in 6/28 chordomas of the extension series (21%; see ‘Methods’). In two of the chordoma cell lines, gains of *T* were found (Supplementary Table 3; Supplementary Fig. 2).

### Recurrent mutation of PI3K signalling genes

An observation of potential therapeutic relevance was the presence of driver events in PI3K signalling genes in our cohort in 17/104 (16%) cases. These included activating mutations in *PIK3CA* (*n* = 9) and truncating variants in *PIK3R1* (*n* = 1) and *PTEN* (*n* = 7). Given the poor prognosis of chordoma, our findings provide a rationale for exploring the utility of targeting PI3K signalling in the treatment of chordoma. Of note in this context, inhibition of mTOR (mammalian target of rapamycin), a downstream node of the PI3K signalling cascade, has shown promising activity in animal models and in some patients^16–18^.

### Driver events in chromatin modelling genes

We identified driver events in further cancer genes not previously implicated in chordoma including recurrent mutation of the SWI/SNF complex sub-unit gene *ARID1A* (4/104 cases). Drivers in another SWI/SNF gene, *PBRM1* (10/104 cases) and the histone methyltransferase, *SETD2* (5/104) implicate defective chromatin modelling as a major driver of chordoma. In 18/104 (17%) tumours, we found at least one driver variant in one of the three genes, with *PBRM1* being one of the most commonly mutated cancer genes in chordoma. It is interesting to note that the contribution of *SETD2, ARID1A* and *PBRM1* to chordoma is reminiscent of the role of these cancer genes in renal cell carcinoma^19^. Known somatic changes of chordoma, such as homozygous deletion of *CDKN2A* ^4^, were recapitulated in our study.

### Recurrent mutation of the *LYST* gene

We next searched for novel genes operative in the pathogenesis of chordoma. We found recurrent truncating mutations in the gene encoding the lysosomal trafficking regulator protein, *LYST* ^20^. In the discovery cohort, five tumours (14%) harboured a single *LYST* mutation each. These mutations comprised three frameshift indels, one nonsense substitution and one disruptive intragenic rearrangement (Fig. 1b; Supplementary Fig. 3).

The apparent enrichment of truncating *LYST* mutations may represent a chance observation, or may be a consequence of positive selection for driver variants. Enrichment by chance would seem unlikely, as we were able to validate this observation. In the extension cohort, we found *LYST-*truncating mutations in 5/67 cases (7%). Here, the frequency of tumours harbouring truncating *LYST* mutations was significantly enriched compared to 4947 non-chordoma tumours (*p* = 2.7 × 10^−6^; Fisher’s exact test; see ‘Methods’). In addition, the pattern of indels observed in *LYST* differed significantly from the expected pattern, including the absence of in-frame indels (*p* = 0.014; permutation test, Supplementary Fig. 4). We next considered that truncating *LYST* mutations may have accumulated as a consequence of hypermutation of the *LYST* locus in chordoma. However, comparing the frequency of *LYST* substitutions and indels in chordoma with breast cancers genomes, we found no evidence of increased mutability of the *LYST* locus in chordoma (see ‘Methods’). Thus, neither chance nor hypermutation would explain the enrichment of truncating mutations in *LYST*. It would therefore seem likely that *LYST* operates as a cancer gene through loss of function mutations. We did not see evidence of biallelic inactivation via a second point mutation or genomic deletion (LOH) in any of the samples bearing a truncating *LYST* mutation. This would suggest that *LYST* is not a classic two-hit tumour suppressor gene. Clearly, additional genomic studies and functional investigations are required to clarify the role of *LYST* in chordoma pathogenesis. Given the apparent specificity of truncating *LYST* mutations to chordoma, they may have utility as an adjunct diagnostic tool.

## Discussion

Here, we presented a comprehensive exploration of the somatic changes that underpin chordoma. Overall, it reveals a relatively quiet cancer genome driven by a limited repertoire of cancer genes.

A key finding of our study was a recurrent somatic duplication of *T*, organised as highly focal single copy-number gains akin to germline alterations of *T* underlying familial chordoma. Whole-genome sequences, which inform on copy number, variant allele frequencies of SNPs and breakpoints at base-pair resolution, now provide clear evidence of focal somatic *T* gains in chordoma. It seems possible that the frequency of *T* duplication has been previously underestimated due to the limited sensitivity of assays utilised, and subtle pattern of mutation. Interestingly, *T* duplications do not evolve into high-level amplifications. This may indicate that an exact gene dosage is required for the development of chordoma.

Our observation of non-random enrichment of truncating mutations in the *LYST* gene indicates that it may operate as a cancer gene in chordoma. Although this proposition requires further validation, one may speculate on the biological role of *LYST* in chordoma development. It may lie in the function of *LYST* as a lysosomal regulator gene^20^. It has been shown that lysosomes are required for notochordal development^21^. Interestingly, lysosomes are the histological hallmark of the ‘physaliphorous’, vacuole-packed cells that characterise chordoma^22^. The functional link of *LYST* to chordoma development may thus lie in aberrant function of notochordal lysosomes, potentially interfering with notochord cell differentiation.

In the context of a quiescent and monotonous driver landscape of chordoma, we were unable to detect a single plausible driver variant in 47/104 tumours, including four tumours interrogated by whole-genome, transcriptome and re-sequencing of known cancer genes at high depth. Genomic studies of larger cohorts may be required, including a detailed exploration of the epigenome, to complete the picture of the pathology of chordoma.

## Methods

### Patient samples

Informed consent was obtained from all subjects and ethical approval obtained from Cambridgeshire 2 Research Ethics Service (reference 09/H0308/165). Patient samples were also obtained from the Stanmore Musculoskeletal Biobank, a satellite of the UCL/UCLH Biobank (HTA Licence Number 12055), which was approved by the National Research Ethics Committee (reference 15/YH/0311). This specific study was approved by the NREC-approved UCL/UCLH Biobank Ethical Review Committee (reference EC17.14).

### Sequencing

Whole-genome, RNA or exome sequencing was performed using the Illumina HiSeq 2000 or 2500 platform. RNA libraries were prepared by enriching for mRNA through poly(A) capture^14^. Exome sequences were selected for using by bait capture (Agilent)^23^. Targeted sequencing was performed by enriching for genomic areas of interest using a custom-made bait capture set (Agilent). Tumour DNA and RNA were extracted from fresh-frozen tissue, which had been reviewed by a pathologist, using standard methods. Normal-tissue DNA was derived from adjacent normal tissue or blood samples.

### Variant calling

The variant calling pipeline of the Cancer Genome Project, Wellcome Trust Sanger Institute, was used to call substitutions (CaVEMan algorithm), indels (Pindel algorithm), structural rearrangements (BRASS algorithm) and copy-number changes (ASCAT algorithm)^14^. The validity of variant calling through this pipeline has previously been established^14^. Note that we included rearrangement calls only if they were validated by definition at base-pair resolution. RNA reads were searched for gene fusion using three different algorithms (TopHat-Fusion^24^; STAR-Fusion (github.com/STAR-Fusion/STAR-Fusion/wiki); deFuse^25^).

### Driver analysis of mutations

We used previously described methods to call driver events^14^. In brief, drivers in recessive cancer genes were called if mutations truncated the gene footprint. In oncogenes, point mutations were considered to be drivers if they mutated previously curated hotspots. Copy-number gains were regarded oncogenic when the copy number of oncogenes was increased to 5 or 9 copies in diploid and tetraploid samples, respectively. Copy-number segments with homozygous deletions or amplifications had to be focal, defined as < 1 Mb in size. The requirement for focality was relaxed for bonafide recessive cancer genes that presented with both focal and larger homozygous deletions.

### Driver analysis of mutations in cell lines

Publicly available whole-genome sequencing reads of five chordoma cell lines were downloaded from www.chordomafoudation.org and processed through our pipeline, as described above. As no matched normal-tissue sequences were available, calling driver events had to be confined to canonical driver mutations. This included copy-number variants that were called manually due to the lack of matched normal-tissue sequences, by inspection of genes of interest.

### Analysis of targeted sequencing data for *T* gains

We developed a method to call copy-number changes from targeted sequencing data (github.com/constantAmateur/TargetedCN/) combining sequence coverage data, allele frequencies of heterozygous SNPs as well as statistically inferred haploblocks. Here, we applied this method to call copy-number changes in *T* from targeted sequences of the whole footprint of *T* plus adjacent intergenic regions. The performance of the method was tested in data drawn from two separate experiments: the target enrichment bait capture set used in this experiment and a second bait set enriching for different genes in different tumour types (angiosarcoma; breast cancer; chondromyxoid fibroma; osteosarcoma; see Supplementary Data 1). We assessed the precision of our method, benchmarked against copy-number analyses from whole-genome sequencing, in determining the copy number of genes interrogated by targeted sequencing in their entirety. Looking at 76 copy-number calls collected from 20 tumours and 8 genes, we found that our method precisely determined copy number in 70/76 calls. Focusing on *T* copy-number calls in chordoma, our method identified 2 of 3 duplications. Thus, our method, although specific, is less sensitive compared with the whole-genome sequencing in calling copy-number changes in genes, including *T*, interrogated in their entirety by targeted sequencing.

The sensitivity of our method depends on the presence of heterozygous SNPs. Accordingly, tumours with no or few heterozygous SNPs in *T* are not informative. Thus, depending on which SNP threshold is chosen, the prevalence of *T* duplications in chordoma changes as follows: no threshold—8/67 cases harboured *T* gains; >20 heterozygous SNPs—6/43 cases harboured *T* gains; > 40 SNPs 6/28 cases harboured *T* gains; > 60 SNPs 4/12 cases harboured *T* gains. On the basis of these analyses, we chose a threshold of > 40 SNPs to define a tumour cohort of informative cases.

### Extraction of substitution signatures

Substitution signatures were extracted by using non-negative matrix factorisation, as implemented in a published algorithm^15^.

### Statistical analyses

The frequency of chordomas harbouring truncating *LYST* mutations was assessed as follows: Exome-sequencing reads of non-chordoma tumours were obtained from The Cancer Genome Atlas and processed by the same analysis pipeline as used for our chordoma study. In this comparison, only coding mutations were considered. Other than a SumMS score of 250 or greater for Pindel calls, no additional post-processing was applied to mutation calls. Overall, 4947 non-chordoma tumours were included in the analysis. Cases that were excluded had poor quality sequencing data or were hypermutators compared with chordoma (defined as ≥100 coding substitutions per exome). The frequency of tumours harbouring a truncating LYST mutation (nonsense, essential splice, start lost, nonsense, out of frame indel) was significantly increased in chordoma (5/67 tumours) versus non-chordoma tumours (13/4947 tumours), as assessed by the Fisher’s exact test. To assess the mutability of *LYST*, we compared the rate of LYST subs and indels in our chordoma genomes to those from a large sample of primary breast cancers (*n* = 229) from a published study^14^. We tested both subs and indels independently for evidence of an increased mutation rate in chordoma using a one-sided Fisher’s exact test followed by multiple hypothesis correction.

### Data availability

Sequencing data have been deposited at the European Genome-Phenome Archive (http://www.ebi.ac.uk/ega/, which is hosted by the European Bioinformatics Institute; Accession Numbers EGAS00001000892 and EGAS00001000895). Chordoma cell line sequencing data are available at www.chordomafoundation.org.

## Electronic supplementary material

Supplementary Information

Description of Additional Supplementary Files

Supplementary Data 1

Supplementary Data 2

Supplementary Data 3

Supplementary Data 4

Supplementary Data 5
