# Supplementary material for: The driver landscape of sporadic chordoma
Source: Nat Commun. 2017 Oct 12;8:890. doi: 10.1038/s41467-017-01026-0 (PMC5638846; doi:10.1038/s41467-017-01026-0)
Supplement: Supplementary file 1 — Supplementary Information [file 41467_2017_1026_MOESM1_ESM.pdf]

Supplementary Figure 1. Copy number of *T* derived from targeted sequencing.

Two examples of tumours that harbour gains in *T*. PD22326a is an example of a highly informative tumour in which a large number of heterozygous SNP confirm the gain in copy number which increased coverage of *T* shows. PD22310a is an example of tumour in which copy number gain was mainly derived from coverage data. In this particular example the call is further substantiated by steps indicating the presence of breakpoints. The plots show coverage (tumour/normal panel) and the B-allele frequency (BAF) of T in each of the tumours. Y-axis of tumour/normal plot: coverage (unit: number of 5' ends of fragments in a bin / (length of bin × reads for sample) × 3×109). The error bars show the 95% confidence interval for the true mean coverage in each coverage bin. The colour coding of the error bars alternates at the boundary of each separately defined capture region. Y-axis of BAF track: B-allele frequency; colours represent different alleles, as inferred statistically from haploblocks of the 1000 genome project.

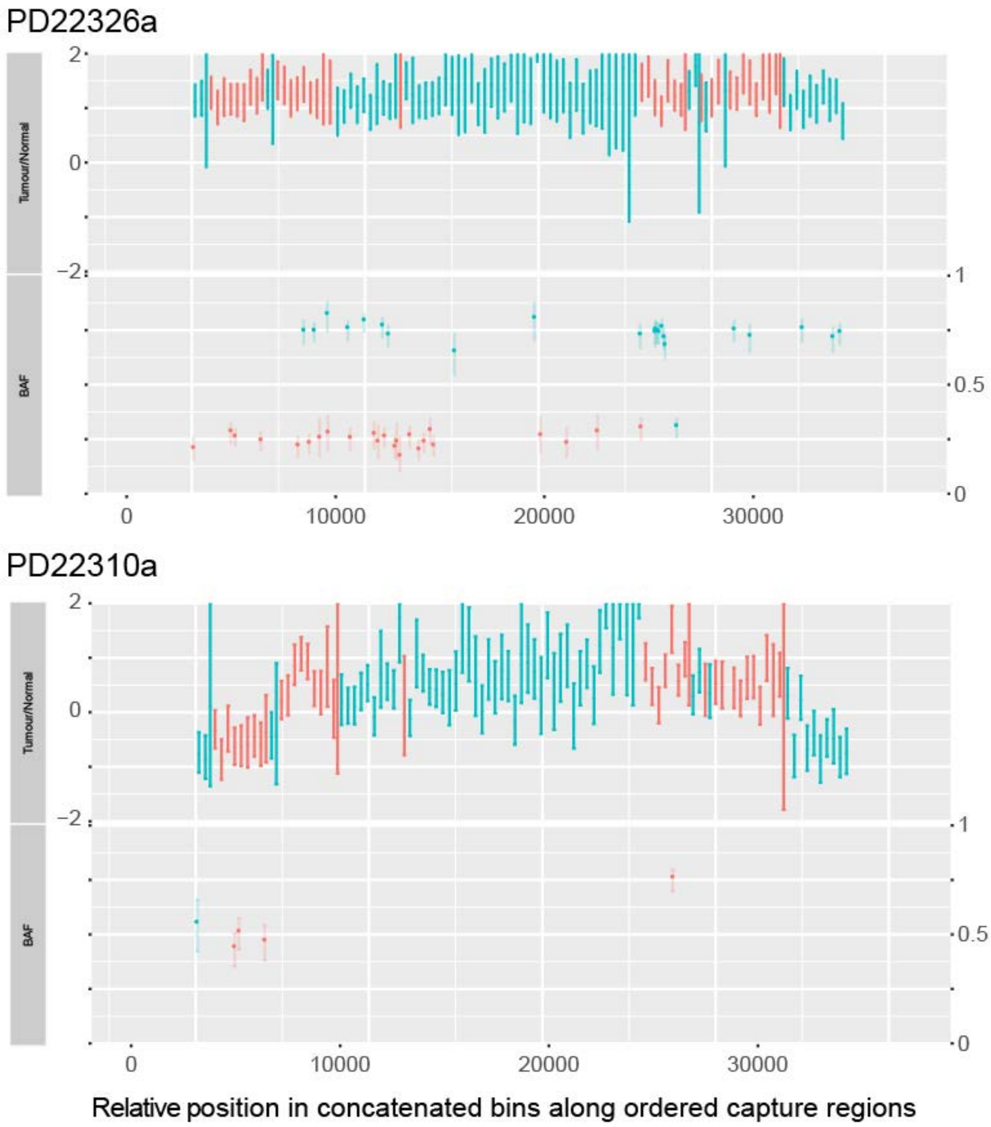

Supplementary Figure 2. Copy number of *T* in chordoma cell lines.

Shown is the raw coverage data for each cell line (Y-axis) along chromosome 6 (x-axis). Two lines harbour copy number gains in *T* (yellow line). Note that in U-CH2 the telomeric portion of the chromosome arm that harbours *T* (6p) is duplicated.

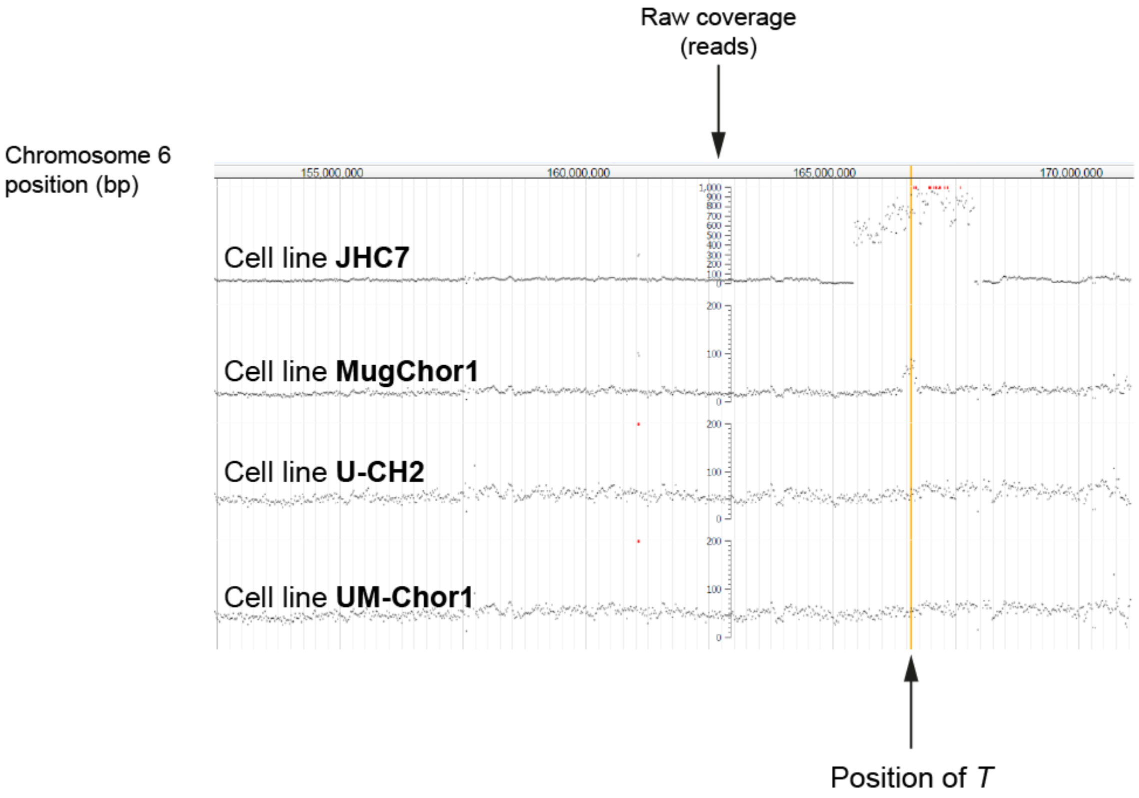

Supplementary Figure 3. Intragenic rearrangement in *LYST* in PD3820a.

Shown is the coverage of *LYST* in this tumour obtained in the targeted sequencing experiment (Y-axis). X-axis: chromosome 1 position in basepairs. Red line: footprint of the *LYST* gene. Vertical black line indicate the position of the breakpoints connected by the arc. Numbers next to line - exact coordinate of breakpoint.

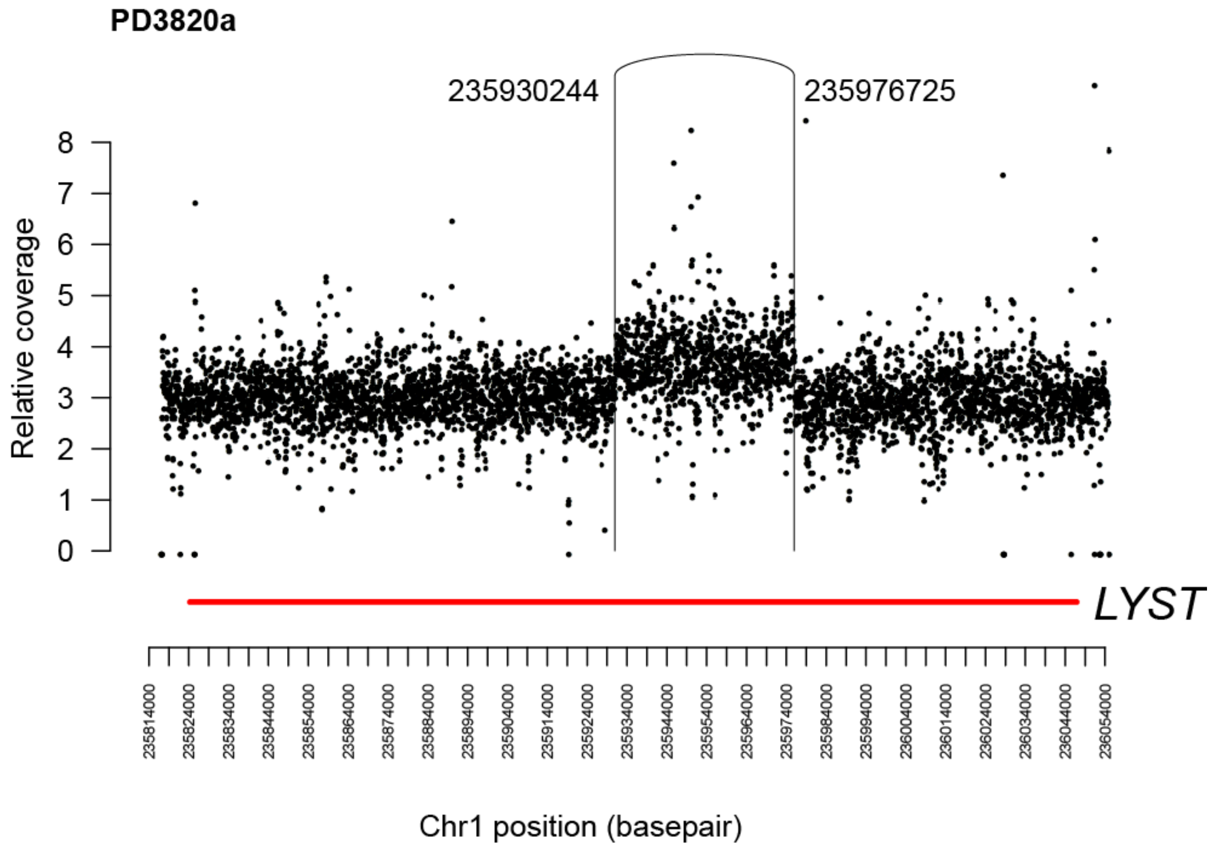

Supplementary Figure 4. Indel size distribution of *LYST* indels compared to diffferent tumour types.

For each tumour type (colour coded bars) the percentage (Y-axis) of indels of a particular size (X-axis; in basepairs) is shown. Coding indels in LYST are shown in torquoise. The figure shows that *LYST* indels do not follow a ‘conventional’ pattern. Breast cancer genomes (n=229) were taken from a previous publication. The osteosarcomas are 37 whole genomes (Behjati *et al.*, Nat Commun. 2017;8:15936). The chordoma genomes are the 11 genomes presented here. For the stastistical analysis for the size distribution of the indels, the indel data was pooled and the assignment randomised to LYST and non-LYST. For each random allocation the Kolmogorov-Smirnov statistic was determined. The resulting distribution of KS statistics was the compared to the observed one to determine whether the KS stastitic occured by chance. Accordingly, the size distribution of *LYST* indels was unlikely to have occured by chance (p=0.014).

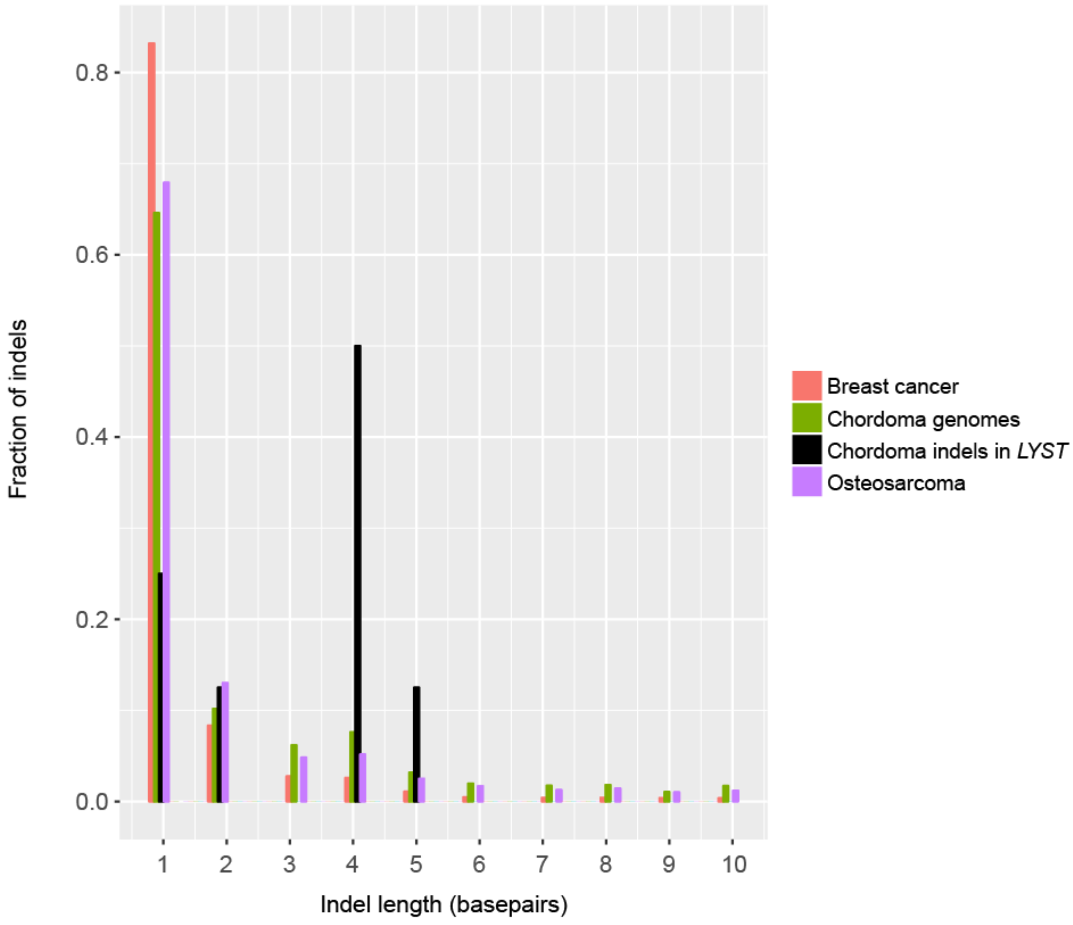

Supplementary Table 1. Driver events in chordoma cell lines.

	T	***CDKN2A***	***TP53***	***PTEN***
**UM-Chor1**	-	-	-	PTEN,L139*
**U-CH2**	*	Homozygous deletion	-	-
**JHC7**	Amplifaction (~1.5 MB)	CDKN2A,P114T	TP53,R213L	-
**Mug-Chor1**	Duplication (~400 kb)	Homozygous deletion	Deletion or homozygous deletion	-

*In this line the telomeric portion of the chromosome arm that harbours *T* (6p) is duplicated.

Supplementary Table 2. Structural rearrangements underlying copy number gains in *T*.

Tumour	Lower_chr	Lower_orientation	Lower_coordinate	Higher_chr	Higher_orientation	Higher_coordinate	Rearrangement_type	Non_template_sequence OR microhology_sequence
PD13479a	6	-	166524986	6	-	166598010	Tandem_duplication	AT
PD13483a	6	+	166513203	6	-	166609453	Inversion	-
PD13483a	6	+	166513535	6	+	166608225	Deletion	T
PD13483a	6	-	166512685	6	-	166608686	Tandem_duplication	T
PD13483a	6	-	166509898	6	-	166514512	Tandem_duplication	CAT
PD13483a	6	-	166473460	6	-	166608758	Tandem_duplication	AGAG
PD13483a	6	-	166511855	6	+	166608301	Inversion	GTT
PD13483a	6	-	166509114	6	+	166513110	Inversion	A
PD11411a	6	-	166513179	6	-	166581695	Tandem_duplication	CTT

Supplementary Table 3. Validation targets and tumours for copy number calling method.

		Bait set 1						Bait set 2	
	Gene >	*LAMA3*	*LYST*	*CDKN2A*	*SOX5*	*SOX6*	*T*	*PTPRB*	*SHPRH*
Tumour	Tumour type								
PD7189a	Spindle cell sarcoma	-	-	-	-	-	-	1	1
PD9056a	Osteosarcoma	-	-	-	-	-	-	1	1
PD11420a	Chordoma	1	1	1	1	1	1	-	-
PD11424a	Chordoma	1	1	1	1	1	1	-	-
PD13455a	Chordoma	1	1	1	1	1	1	-	-
PD18735a	Chordoma	1	1	1	1	1	1	-	-
PD11411a	Chordoma	1	1	1	1	1	1	-	-
PD11421a	Chordoma	1	1	1	1	1	1	-	-
PD13479a	Chordoma	1	1	1	1	1	1	-	-
PD13483a	Chordoma	1	1	1	1	1	1	-	-
PD18817a	Chordoma	1	1	1	1	1	1	-	-
PD7190a	Osteosarcoma	-	-	-	-	-	-	1	1
PD7191a	Spindle cell sarcoma	-	-	-	-	-	-	1	1
PD7514a	Chondromyxoid fibroma	-	-	-	-	-	-	1	1
PD7515a	Chondromyxoid fibroma	-	-	-	-	-	-	1	1
PD8618a	Breast cancer	-	-	-	-	-	-	1	1
PD8622a	Breast cancer	-	-	-	-	-	-	1	1
PD7188a	Angiosarcoma	-	-	-	-	-	-	1	1
PD7192a	Angiosarcoma	-	-	-	-	-	-	1	1
PD8623a	Breast cancer	-	-	-	-	-	-	1	1
