# Supplementary material for: The driver landscape of sporadic chordoma
Source: Nat Commun. 2017 Oct 12;8:890. doi: 10.1038/s41467-017-01026-0 (PMC5638846; doi:10.1038/s41467-017-01026-0)
Supplement: Supplementary file 2 — Description of Additional Supplementary Files [file 41467_2017_1026_MOESM2_ESM.pdf]

Description of Additional Supplementary Files

File Name: Supplementary Data 1
Description: Cancer genes screened by targeted sequencing

File Name: Supplementary Data 2
Description: Overview of 104 chordomas

File Name: Supplementary Data 3
Description: Coding mutation in 37 chordomas (11 genomes and 26 exomes)

File Name: Supplementary Data 4
Description: Substitutions and indels in 11 chordoma genomes

File Name: Supplementary Data 5
Description: Structural rearrangements found in 11 chordoma genomes
